# Tumor Necrosis Factor Alpha-308G/A Polymorphism and the Risk of Multiple Myeloma: A Meta-Analysis of Pooled Data from Twelve Case-Control Studies

**DOI:** 10.4274/tjh.galenos.2019.2018.0238

**Published:** 2019-05-03

**Authors:** Yingchao Li, Yong Lin

**Affiliations:** 1 Xiamen University Zhongshan Hospital, Department of Orthopedics, Xiamen, China; 2 Xiamen University Zhongshan Hospital Clinical Laboratory, Xiamen, China; 3 Medical College of Xiamen University, Institute of Infectious Disease, Xiamen, China

**Keywords:** Multiple myeloma, Tumor necrosis factor-α, Polymorphism, Meta-analysis

## Abstract

**Objective:**

Tumor necrosis factor alpha (TNF-α) is an important cytokine involved in inflammation, immune response, and other biological processes. The association between polymorphism -308G/A in its promoter and the risk of multiple myeloma (MM) is not clear. Thus, we conducted a meta-analysis to clarify this question.

**Materials and Methods:**

Twelve eligible studies, which included 2204 MM cases and 3478 controls, were enrolled in our meta-analysis by searching the PubMed, China National Knowledge Infrastructure, Scopus, Web of Science, and Google Scholar databases up to December 2018. The effect of polymorphism -308G/A on MM risk was evaluated by calculating the pooled odds ratio (OR) and the 95% confidence interval (CI). Furthermore, the Q-test and I2 statistical analyses were used to estimate the degree of heterogeneity. Sensitivity analysis was conducted to test the robustness of the meta-analysis results. Publication bias was assessed by Egger’s test and visual inspection of a funnel plot.

**Results:**

In the dominant model, -308G/A polymorphism was associated with reduced MM risk (OR=0.80, 95% CI: 0.65-0.97), and it also demonstrated a significant protective effect with a pooled OR of 0.82 (95% CI: 0.68-0.99) in the Caucasian subgroup. Because of the limited number of individual studies with AA genotype carriers, only eight studies were included in the recessive model, and no significant difference was observed. Moreover, the meta-analysis of the allele frequency demonstrated that the A allele has a protective effect against MM risk with a pooled OR of 0.83 (95% CI: 0.69-0.99). Sensitivity analysis suggested that the synthesized effect size was not influenced by any individual study. Moreover, the Egger’s test statistical analysis suggested that publication bias was not obvious in the present analysis.

**Conclusion:**

Overall, the -308G/A polymorphism was associated with reduced MM risk in the dominant model and allele frequency. Further investigation is needed to gain better insight.

## Abstract (tr)

**Amaç:**

Tümör nekroz faktör alfa (TNF-α) enflamasyon, immün cevap ve diğer biyolojik süreçlerde rol alan önemli bir sitokindir. TNF-α -308G/A promotor bölge polimorfizmi ile multipl myelom (MM) riski arasındaki ilişki net değildir. Bu soruya cevap aramak amacıyla bir meta-analiz çalışması gerçekleştirdik.

**Gereç ve Yöntemler:**

Meta analize 2018 Aralık ayına kadar PubMed, China National Knowledge Infrastructure, Scopus, “Web of Science”, ve “Google Scholar”da yayınlanmış olan 2204 MM hastası ve 3478 kontrol içeren 12 çalışma dahil edildi. -308G/A polimorfizminin MM üzerine olan etkisi birleştirilmiş odds oranı (OR) ve %95 güven aralığı (CI) ile değerlendirildi. Heterojenite derecesinin hesaplanması için Q-test ve I2 istatistiksel analizleri kullanıldı. Meta analiz sonuçlarının kuvvet testi için hassasiyet analizi kullanıldı. Yayın yanlılık değerlendirilmesi Egger testi ve huni grafiğinin görsel incelemesi ile yapıldı.

**Bulgular:**

Baskın modelde, -308G/A polimorfizmi azalmış MM riski ile ilişkili bulundu (OR=0,80, %95 CI: 0,65-0,97), ve bu polimorfizm varlığının anlamlı koruyucu etkisi beyaz ırkta 0,82 (%95 CI: 0,68-0,99) birleştirilmiş OR ile de gösterildi. AA genotip taşıyıcılarının bireysel çalışmalarda daha az sayıda bulunması nedeniyle çekinik modele sadece 8 çalışma dahil edildi ve anlamlı bir farklılık gözlenmedi. Dahası, allel frekansının meta analizinde A allelinin MM için koruyucu etkisi 0,83 (%95 CI: 0,69-0,99) birleştirilmiş OR ile gösterildi. Hassasiyet analizi sentezlenen etki büyüklüğünde hiçbir çalışmanın tek başına belirleyici etkisinin olmadığını gösterdi. Ayrıca, Egger test istatistiksel analizi ile bu çalışmada yayın yanlılığının olmadığını ortaya kondu.

**Sonuç:**

Sonuç olarak, -308G/A polimorfizmi baskın modelde ve allel sıklığında MM riskinde azalma ile ilişkili bulunmuştur. Bu ilişkinin daha iyi anlaşılabilmesi için ileri çalışmalar gereklidir.

## Introduction

Multiple myeloma (MM) is a bone marrow-based disseminated neoplasm commonly preceded by premalignant monoclonal gammopathy of undetermined significance [1]. Among the hematologic malignancy types, MM accounts for approximately 10% of diagnosed cases, maintaining the second position after non-Hodgkin’s lymphoma [2]. According to the latest statistics, there are 30,330 new MM cases and 12,650 deaths attributed to MM in the United States annually [3]. With the rapid progress made in pharmaceutical research, novel proteasome inhibitors and immune modulatory drugs have been applied in the treatment of MM [4], and the prognosis of MM has significantly improved among all MM patients. Despite the improvement in both treatment and survival, MM is currently regarded as an incurable disease, and the major goal of treatment is to achieve partial or complete remission. Therefore, it is of critical importance to investigate the risk factors of MM and to identify high-risk populations at the early stage of the disease.

Previous studies have revealed that genetic abnormalities contribute to the risk of developing MM, especially genes related to the immune response. For example, the rs2285803 polymorphism located in the human leukocyte antigen (HLA) region was found to be associated with an elevated MM risk in the European population [5] but not in Chinese individuals [6]. Moreover, a study identified several polymorphisms in HLA by applying a novel statistical model, suggesting the important role of HLA in MM [7]. Previous studies have also suggested that mutations in germline lysine-specific demethylase 1 and elongation factor for RNA polymerase II 2 (ELL2) are associated with an elevated risk of MM [8,9]. Tumor necrosis factor alpha (TNF-α) is an important cytokine, and cytotoxin triggers have been implicated in tumor regression, septic shock, and cachexia [10]. Mutations in the promoter region of TNF-α may affect the binding of transcription factors and consequently result in alterations in mRNA expression. The -308G/A polymorphism of TNF-α has been widely investigated in relation to various diseases, including infectious diseases and cancers. The A allele of the -308G/A polymorphism is associated with stronger transcription activity compared with the wild type and increased TNF-α expression in vivo [11]. Moreover, most HLA variations associated with MM can be explained by rs2285803, and we found that the -308G/A polymorphism and rs2285803 are in linkage disequilibrium with a D’ value of 0.7308 and an R^2^ value of 0.0197. Considering the findings that we observed, in the present study, we conducted a meta-analysis on the -308G/A polymorphism and MM risk in accordance with the Preferred Reporting Items for Systematic Reviews and Meta-Analyses guidance, which would provide information on the association between polymorphisms in both TNF-α and HLA and MM.

## Materials and Methods

### Search Strategy and Study Eligibility

A literature search was independently conducted by two investigators for genetic studies on TNF-α in PubMed, the China National Knowledge Infrastructure (CNKI), Scopus, Web of Science, and Google Scholar databases without any restriction of publication language. All relevant studies reported up to 10 December 2018 and the following key words were searched: “multiple myeloma”, “plasma cell”, “plasmacell”, “plasmacytoma”, “myelomatosis”, “Kahler’s disease”, “TNF-α”, “tumor necrosis factor alpha”, and “-308G/A”.

Studies that fulfilled all the following criteria were included in the meta-analysis: (1) studies used case-control study design; (2) studies evaluated the association between TNF-α polymorphisms and the risk of developing MM; and (3) studies included genotype distribution of TNF-α polymorphisms in both cases and controls and other essential information required to estimate the odds ratio (OR) and 95% confidence interval (CI). Studies that met any one of the following criteria were excluded: (1) data were not relevant to the association between TNF-α polymorphisms and MM risk; (2) reviews, cases reports, editorial comments, and communications were included; or (3) there were insufficient data to estimate OR and 95% CI.

### Quality Assessment and Data Extraction

The Newcastle-Ottawa Scale was used to evaluate the quality of the enrolled studies independently by two investigators. Disagreements between the two investigators were settled by discussions to reevaluate the methodological quality of original studies. 

The extraction of data from individual studies included the following: the surname of the first author, the year of publication, the location of the study, the ethnicity and the source of controls, the genotyping method, the Hardy-Weinberg equilibrium (HWE) in controls, and the count of each TNF-α genotype in MM cases and controls.

### Statistical Analysis

The strength of the association between TNF-α polymorphism and MM risk was evaluated by OR and 95% CI. We applied the following models to calculate different ORs: the dominant genetic model (GA+AA vs. GG), the recessive genetic model (AA vs. GA+GG), and the allele model (A vs. G). Z-tests were used to determine the statistical significance of pooled ORs. The heterogeneity between enrolled studies was assessed by using the Q-test, and we applied a random-effects model to calculate pooled effect size. Subgroup analysis by ethnicity was performed to estimate and demonstrate the pooled MM risk caused by TNF-α polymorphism in different races. Each study was removed in turn for sensitivity analyses, and the remaining studies were reanalyzed to assess the stability of the results. Moreover, publication bias among enrolled studies was examined using Egger’s test, where a p-value of less than 0.10 was considered statistically significant. Meta-regression was used to identify the source of heterogeneity among covariates in the presence of heterogeneity. If the intercept was significantly different from zero, the estimate of the effect was considered biased. All statistical analyses were performed with STATA Version 12.0 software (Stata Corp, College Station, TX, USA). All p-values in the present study were two-sided, and p< 0.05 was considered statistically significant unless otherwise specified.

## Results

### Study Identification and Main Characteristics

**Figure 1:**
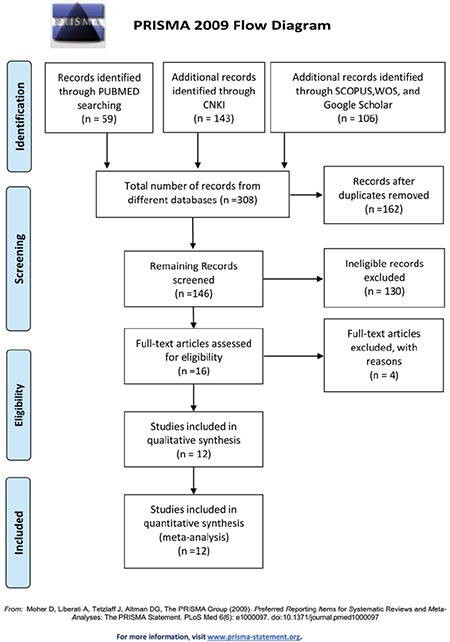
Preferred Reporting Items for Systematic Reviews and Meta-Analyses (PRISMA) flow diagram for inclusion and exclusion of studies in the meta-analysis. CNKI: China National Knowledge Infrastructure.

In total, we identified 167 records from the PubMed and CNKI databases in a primary literature search, and after removing duplicates, 135 records were subjected to title and abstract screening. Sixteen of them were reviewed in full; two were removed because of the absence of genotype distribution data, and two were excluded due to possible overlapping subjects (Figure 1). Finally, 12 studies met the inclusion criteria for our meta-analysis for evaluating the relationship between the -308G/A polymorphism and MM risk [12,13,14,15,16,17,18,19,20,21,22,23]. Among these studies, eight were based on Caucasian populations, and the remaining four were conducted with Asian subjects. The quality score average was 7.3, which combined all the enrolled studies together; a score greater than 5 was considered appropriate for inclusion in the meta-analysis. Based on the chi-square test results, the genotype distribution in the control group was consistent with the HWE among all 12 enrolled studies. The name of the first author, year of publication, country, ethnicity, source of controls, genotyping method, and HWE in the control group are listed in Table 1. The quality score of each individual study is shown in Table 2 and the detailed genotype distribution is demonstrated in Table 3.

### Quantitative Synthesis

**Figure 2:**
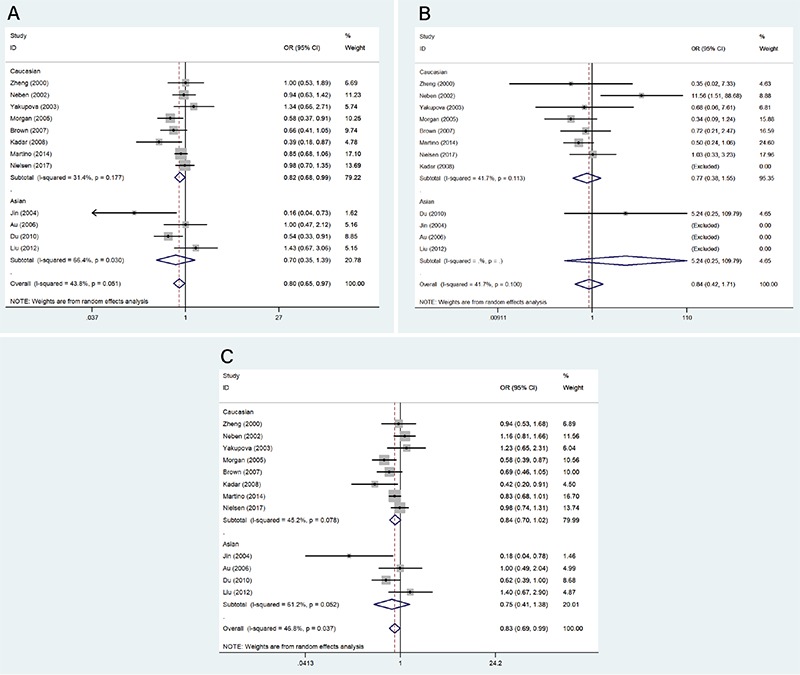
The association of tumor necrosis factor alpha -308G/A polymorphism with multiple myeloma risk in (A) dominant model, (B) recessive model, and (C) allele model. OR: Odds ratio, CI: confidence interval.

The genotype distribution of the -308G/A polymorphism in the cases and controls of all enrolled studies was extracted, and based on that, we performed a meta-analysis; the main outcome is demonstrated in Figure 2 and Table 4. In the dominant model, the -308G/A polymorphism was associated with a reduced MM risk (OR=0.80, 95% CI: 0.65-0.97). The subgroup analysis showed a similar association between the -308G/A polymorphism and MM risk in the Caucasian (OR=0.82, 95% CI: 0.68-0.99) but not the Asian subgroups (OR=0.70, 95% CI: 0.35-1.39). Due to the very limited number of AA homozygous carriers, only one Asian study was included in the recessive model, and the overall effect size based on eight studies was 0.84 (95% CI: 0.42-1.71). For the allele model, according to our estimation, the A allele of TNF-a -308G/A polymorphism confers a protective effect against MM risk with a pooled OR of 0.83 (95% CI: 0.69-0.99) in the overall population, and significance was observed. However, no significant association was observed in the Caucasian (OR=0.84, 95% CI: 0.70-1.02) and Asian (OR=0.75, 95% CI: 0.41-1.38) populations in the estimation of subgroup analysis.

### Heterogeneity Analysis

As seen in the quantitative synthesis, heterogeneity was observed in the overall population and in both subgroups in the dominant model; the p-values of the Q-test were all less than 0.05. Similar to the dominant model, intermediate heterogeneity was detected in the allele model. However, no significant heterogeneity was found in the recessive model according to the Q-test results.

### Sensitivity Analysis

**Figure 3:**
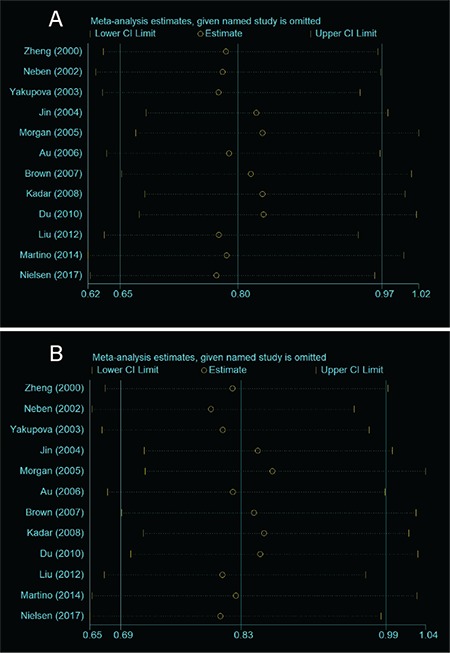
Sensitivity analysis for tumor necrosis factor alpha -308G/A polymorphism in (A) dominant model and (B) allele model. CI: Confidence interval.

To evaluate the robustness of the present meta-analysis, we performed sensitivity analysis by sequentially removing each eligible study and observing the changes in the overall effect size. As shown in Figure 3, the significance of the overall effect size was not influenced by any single study in both the dominant and allele models, indicating that our results were statistically robust.

### Publication Bias

**Figure 4:**
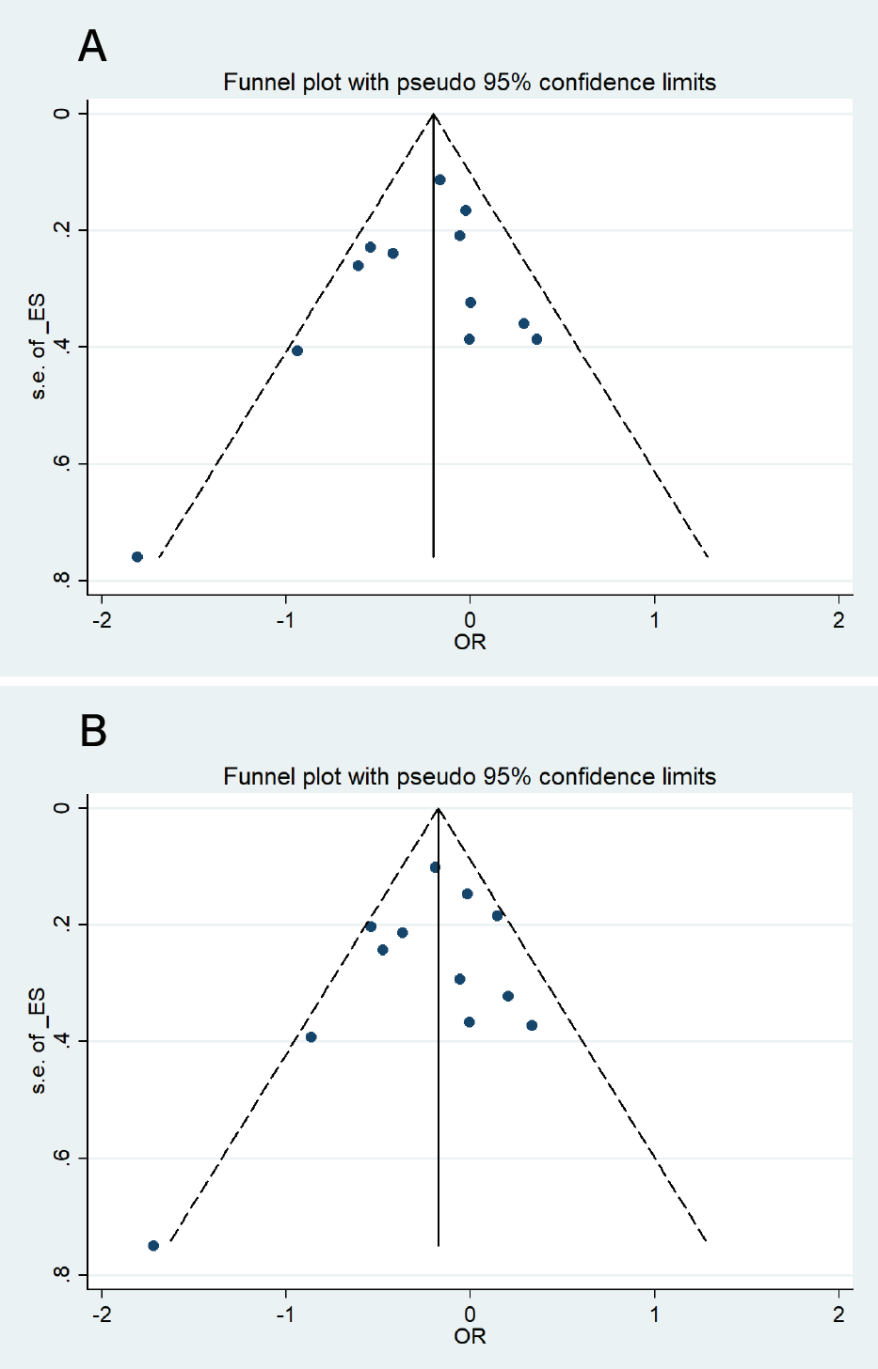
Begg’s funnel plot for the evaluation of publication bias of tumor necrosis factor alpha -α -308G/A polymorphism in (A) dominant model and (B) allele model. OR: Odds ratio.

We applied Begg’s funnel plot and Egger’s test to assess publication bias in the present meta-analysis of the -308G/A polymorphism and MM risk. The funnel plots’ shapes of the dominant and allele models (Table 5; Figure 4) did not provide obvious evidence of asymmetry, and all the p-values of Egger’s test were greater than 0.05, providing statistical evidence for the funnel plots’ symmetry. Thus, the above results suggest that publication bias was not evident in this meta-analysis.

### Meta-regression

As demonstrated in the previous section, heterogeneity was observed in both the dominant and allele model analyses but not in the recessive model. Therefore, meta-regression was conducted to identify the possible source of heterogeneity by testing the year of publication, sample size, ethnicity, study quality, control source, and genotyping method. As can be seen in Table 6, the p-values of all the tested covariates were greater than 0.05, indicating no contribution to heterogeneity.

## Discussion

TNF-α is a proinflammatory cytokine that is mainly secreted by multinuclear giant cells, with a wide range of biological activities, including the regulation of host immune functions and the inflammatory reaction process [24]. Moreover, TNF-α is capable of inducing cell apoptosis, and in contrast, it can accelerate tumor growth. Growing evidence has demonstrated that TNF-α participates in several key processes of tumor progression, including oncogene activation, DNA damage, and tumor metastasis [25]. For instance, it has been reported that TNF-α has elevated expression in colorectal cancer tissue compared with normal colorectal tissue, and that cancer tissues in advanced stages have higher TNF-α expression compared with cancer tissues in earlier stages [26]. As mentioned before, the -308G/A polymorphism is associated with elevated expression of TNF-α mRNA through its effect on transcription. Our meta-analysis comprehensively reviewed published findings and demonstrated that it has a protective effect against MM risk in the dominant model and allele analysis.

Substantial studies have shown that the -308G/A polymorphism is associated with elevated constitutive and inducible protein levels compared with wild-type carriers [27]. A low concentration of TNF-α was observed in subjects carrying the GG genotype, and intermediate and high levels were associated with the GA and AA genotypes, respectively. Therefore, it was reasonable to assume that the -308G/A polymorphism increases the risk of developing MM through its effect on the TNF-α expression level. It has been acknowledged that the inflammation and immune responses triggered by TNF-α lead to the progression of cancer and often predict a worse outcome. A previous study conducted on 44 MM cases demonstrated that MM patients with advanced progression had significantly elevated serum TNF-α levels compared with normal controls [28]. Consistent with this result, Jurisić and Colović [29] examined the TNF-α levels in MM patients and found that serum level positively correlated with clinical stage and osteolysis, which is a severe complication of MM. Moreover, a cell assay revealed that TNF-α was capable of inducing IL-6 expression via the JAK/STAT pathway in U266 MM cells [30]. Thus, both epidemiological observations and laboratory studies support the unfavorable effects of TNF-α on MM. However, our results were inconsistent with the previous findings. 

The pooled OR in the dominant and allele model showed a protective effect of the -308G/A polymorphism. A possible explanation is that inflammation caused by elevated TNF-α would definitely promote the progression and development of MM, which is confirmed by comparing MM cases in different stages. TNF-α has also been proven to possess anti-tumor effects through various mechanisms. For instance, TNF-α has been shown to have cytotoxic activity in tumor cells in paraformaldehyde-fixed activated monocytes [31]. Moreover, it has been revealed that TNF-α is associated with B-cell proliferation and immunoglobulin production by interacting with TNF-R2 in healthy individuals [32]. Based on these findings, we assume that elevated TNF-α levels can reduce the MM risk by improving immune surveillance and eliminating tumor cells, but in individuals who have already developed MM, the elevated TNF-α level may have unfavorable effects associated with shorter survival time.

In subgroup analysis by ethnicity, we observed contradictory results in the dominant model but not allele frequency. As observed, the -308G/A polymorphism has a protective effect in the Caucasian population but not in the Asian population; we assume that this can be attributed to the limited number of Asian population studies when compared with Caucasian populations. Therefore, it is possible that the subgroup analysis showed a null association in the Asian population. In addition, we also analyzed the difference between genotyping methods employed by included studies. Due to the limited number of included studies, we categorized the genotyping methods as polymerase chain reaction-restriction fragment length polymorphism (PCR-RFLP), which is a classic genotyping method, and others, as one subgroup. PCR-RFLP is a technique that detects DNA sequence mutations by the lengths of fragments after digestion with specific restriction endonucleases [33]. However, it requires a larger amount of DNA and often involves multiple manual processes. In addition, techniques such as TaqMan offer rapid processing and a more sensitive way to determine SNPs with distinct fluorescent dye-based technology [34]. The subgroup analysis showed a similar trend between subgroups in both the dominant model and allele analysis. Based on the results, we assume that the genotyping methods did not interfere with the overall effect size.

### Study Limitations

Some limitations of our study must be mentioned. First, the rare frequency of the AA genotype in some of the included studies led to the unavailability of these studies in the recessive model. Second, the present study failed to identify the source of heterogeneity using meta-regression, although heterogeneity was rather intermediate.

Nonetheless, we provided novel insight into the association investigated in this study, suggesting that elevated TNF-α levels may reduce MM risk, which deserves investigation of its underlying mechanism.

## Conclusion

In summary, only intermediate heterogeneity was detected in two genetic models with no sign of publication bias. We attempted to identify the source of heterogeneity by conducting meta-regression; however, no contribution to the heterogeneity was found. The sensitivity analysis indicated that the pooled effect size was not influenced by any single study, indicating satisfactory robustness of the present study. Therefore, we concluded that the synthesized effects and conclusions about TNF-α polymorphism were solid.

## Figures and Tables

**Table 1:**
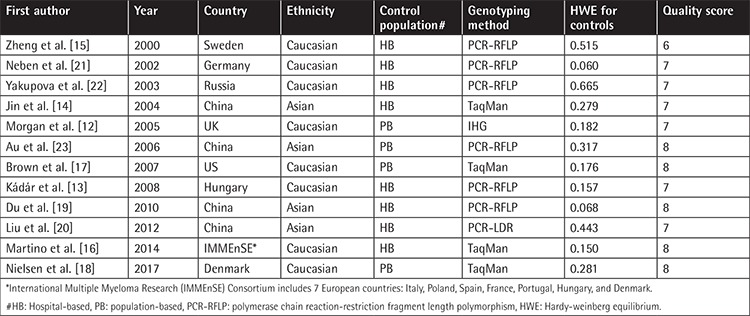
Main characteristics of enrolled studies in meta-analysis.

**Table 2:**
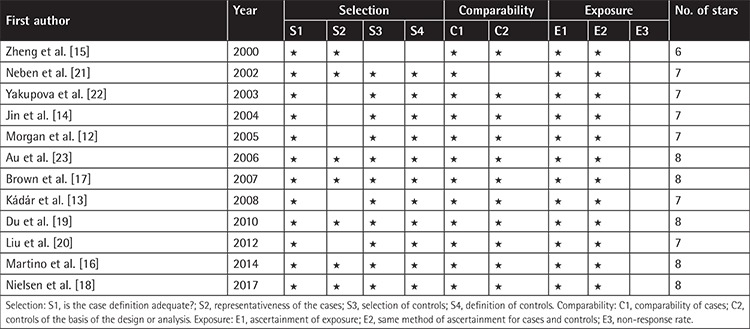
The quality score of included studies by using Newcastle-Ottawa Scale.

**Table 3:**
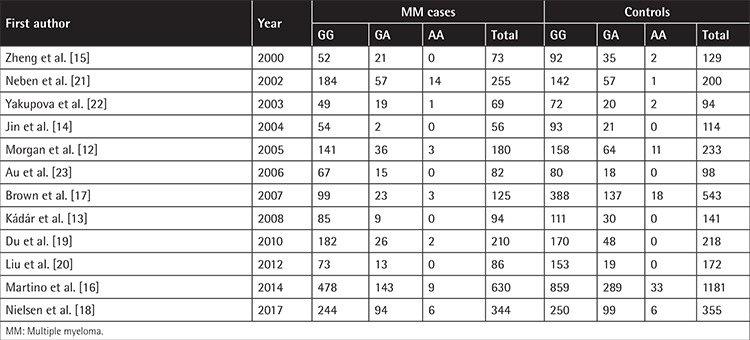
Tumor necrosis factor alpha -308G/A polymorphism genotype distribution in cases and controls.

**Table 4:**
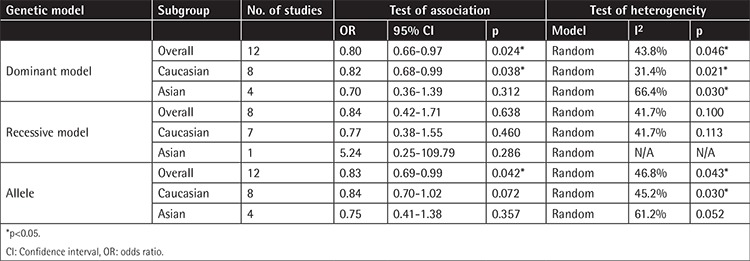
Summary of odds ratios and 95% confidence intervals of tumor necrosis factor alpha -308G/A polymorphism with multiple myeloma risk.

**Table 5:**
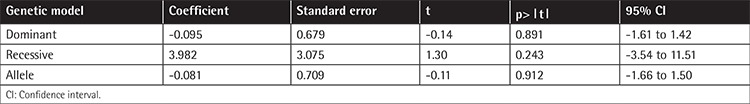
The evaluation of publication bias by using Egger’s test.

**Table 6:**
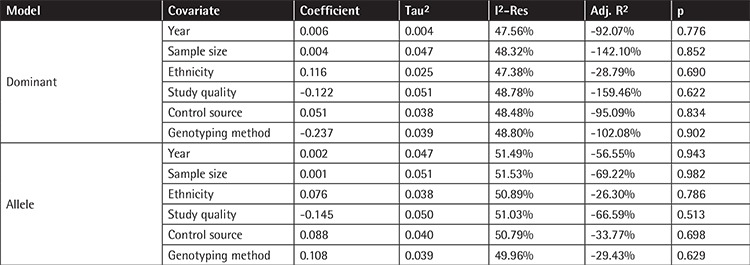
Meta-regression of tumor necrosis factor alpha polymorphism -308G/A polymorphism with multiple myeloma risk.
